# Designing a productive, profitable integrated farming system model with low water footprints for small and marginal farmers of Telangana

**DOI:** 10.1038/s41598-024-66696-5

**Published:** 2024-07-24

**Authors:** Rayapati Karthik, M. Venkata Ramana, Ch. Pragathi Kumari, T. Ram Prakash, M. Goverdhan, D. Saida Naik, Mandapelli Sharath Chandra, M. Santhosh Kumar, Nallagatla Vinod Kumar, L. Peace Raising, Kirttiranjan Baral, Rajan Bhatt, Nadhir Al-Ansari, Khalid M. Elhindi, Mohamed A. Mattar

**Affiliations:** 1 College of Agriculture, Rajendranagar, PJTSAU, Hyderabad, Telangana India; 2 AICRP On Integrated Farming System, Professor JayashankarTelangana State Agricultural University, Rajendranagar, Telangana India; 3 AICRP On Weed Management, Professor JayashankarTelangana State Agricultural University, Rajendranagar, Telangana India; 4 Department of Crop Physiology, Professor JayashankarTelangana State Agricultural University, Rajendranagar, Telangana India; 5 Department of, GBPUA&T, Pantnagar, U.S. Nagar, Uttarakhand 263145 India; 6 Division of Agronomy, IARI, New Delhi, India; 7 PAU-Krishi Vigyan Kendra, Amritsar, Punjab 143601 India; 8 Department of Civil, Environmental and Natural Resources Engineering, Lulea University of Technology, 97187 Luleå, Sweden; 9 Department of Plant Production, College of Food and Agriculture Sciences, King Saud University, P.O. Box 2460, Riyadh, 11451 Saudi Arabia; 10 Department of Agricultural Engineering, College of Food and Agriculture Sciences, King Saud University, P.O. Box 2460, Riyadh, 11451 Saudi Arabia

**Keywords:** Small and marginal farmers, Integrated farming system, Telangana, Sustainable yield index, Water footprint, Sustainability, Plant reproduction

## Abstract

In the years 2021–2022 and 2022–2023, an experiment was carried out at the IFS Unit, College of Agriculture, PJTSAU, Rajendranagar in order to determine the best one-acre integrated farming system model for Telangana's small and marginal farmers. Seven farm models among which six models were developed by combining the various components *i.e., *cropping systems, fruit cropfodder crops and livestock components, in different proportions, and compared with rice-groundnut system which is a major farming approach in Telangana using randomized block design. The seven models were as follows: M1: Rice–Groundnut; M2: Rice–Groundnut, Pigeonpea + Sweetcorn (1:3)—Bajra, Bt cotton + Greengram (1:2)—Maize; M3: Rice–Groundnut, Pigeonpea + Sweetcorn (1:3)—Bajra, Pigeonpea + Maize (1:3)—Sunhemp; Napier grass, Sheep (5 + 1); M4: Rice–Groundnut, Pigeonpea + Sweetcorn (1:3)—Bajra, Bt cotton + Greengram (1:2)—Maize, Pigeonpea + Maize (1:3)—Sunhemp, Poultry unit; M5: Guava, Hedge Lucerne, Napier grass, Bt cotton + Greengram (1:2)—Maize, Sheep (5 + 1); M6: Guava, Bt cotton + Greengram (1:2)—Maize, Rice–Groundnut, Poultry; M7: Rice–Groundnut, Pigeonpea + Sweetcorn (1:3)—Bajra, Pigeonpea + Maize (1:3)—Sunhemp; Napier grass, Hedge lucerne, Poultry (100), Sheep (5 + 1). Based on a 2-year average, the Model M7 system produced 9980 Rice Grain Equivalent Yield(RGEY)kg of output per acre, with gross and net returns of ₹210,439 and ₹124,953 respectively, and recovered a B:C ratio of 2.46. It has recorded highest sustainable yield index (SYI) of 0.673 and value index of 0.772 with the lowest water footprint of 259.0 L/kg. This study reveals that adopting an integrated farming system is the optimal approach for effectively combining productive, financially rewarding, and diversified enterprises within a single acre of land.d. This system should be recommended for maximum benefits to smallto small and marginal farmers in Telangana's southern hills and plateau.

## Introduction

The cultivation of cereal-based crops, which is subject to significant risks of climate anomalies like floods and droughts, arethe focus of Indian farmers, particularly the small and marginal farmers. According to the estimated effects of climate change, grain yield losses could be up to 35% for rice, 20% for wheat, 50% for sorghum, and 60% for maize, which poses a threat to livestock production as this may lead to feed scarcity and climatic abnormalities^1^. Because of these abnormalities, farmers are unable to earn enough money to support their families^2^. In India, more than 85% of farming households with less than a hectare of land comes under small and marginal farmers category who face challenges to finance literacy and education^3^ and this number might soar in the near future as land fragmentation is severely impacting the Indian farming community. Their farms are small and dispersed and most of the inputs have become expensive and out of reach to these resource-poor farmers which exhibited farming as an uneconomic and unsustainable enterprise.

. Additionally, small farmers are less likely to practicemodernfarming because of less investment and risk taking capacity^4^. Conventional agriculture that has been carried out so far has caused various problems including increased costs of energy-based inputs, reduced farm income, economic and ecological problems i.e., poor ecological diversity, soil erosion, and both soil and water pollution. Solving the concerns of small and marginal farmers would help in enhancing the India’s economy and reduce the income inequalities. While various initiatives have aimed to enhance the productivity of different farming system components, a complete integration using the farming system concept has not been achieved. Emphasizing integrated farming systems is essential to address the fundamental requirements of households, encompassing human sustenance (cereals, pulses, oilseeds, milk, fruit, honey, fish flesh, etc.), provision of feed and fodder for animals, and the supply of fuel and fiber for daily needs.

In order to address the different needs of farm households while preserving the resource base and upholding environmental quality, farming systems serve as an effectiveresource management method^5^. In comparison to cropping alone, integrated farming system (IFS) models established in various regions of the country have been proven to greatly boost net profit. These models include dairy, duckery, poultry, horticulture, apiculture, pisciculture, and plantation crops together with crops. Farming systems are more productive overall, less prone to volatility, and produce fewer negative externalities than simplified farms^6^. Farming systems are characterised by the temporal and spatial mixing of crops, livestock, fishery, and allied activities in a single farm^6^. IFS has obtained higher productivity of 32.46 t ha^−1^ and net returns of $3828 which is 1.6 and 3.0 times higher, respectively compared to conventional systems^7^.

The IFS is one of the best options for enhancing the well-being of smallholders and ensuring sustainable livelihoods. It not only enhances the nutritional and economic standing of farming families but also boosts employment opportunities and optimizes the use of agricultural resources. The IFS integrates agricultural and animal enterprises, which is drawing fresh attention from marginal, small, and medium farmers who cultivate less than one hectare^8^. The fundamental principle of integration is that the output of each company should serve as input for another, fostering complementarity among them^9^. Emphasizing enhanced ecosystem functioning, including nutrient recycling, soil formation, and fertility improvement, the IFS strategy advocates for ecological intensification and aims to minimize reliance on anthropogenic inputs^10^. It also enhances the sustainability of the environment^11^. Considering that they gain from business synergies, product diversity, and ecological dependability, effectively managed IFSs are anticipated to be less risky^8^. Swarnam et al.^12^ indicated that livestock imparts greater stability to the overall system, as it was less affected by climatic factors such as floods and water scarcity and strong and positive interlinks between components in IFS has resulted insustainable yield index of 0.89 which is 0.28 in case of traditional system.

Research on IFS facilitates the identification of each component's contributions, particularly in terms of soil sustainability. Given the variations in soil, climatic conditions, and cultural factors across different regions, it is crucial to develop region-specific IFS models. The purpose of this study is to develop a suitable IFS model by the integration of two or multiple components which produce higher yields, income and maintains soil sustainability compared to conventional systems. Cropping systems and livestock meat preferences vary from region to region which indicates the essentiality of location specific IFS models. The present scenario underscores the need to establish a tailored one-acre IFS model that can prove beneficial for small and marginal farmers in Telangana State and other states sharing similar agro-climatic characteristics in India. This studywould help thescientists and policy makers to compare the various IFS combinationsin terms of yields, profits and sustainability withexisting cropping systems of small and marginal farmers and could recommend the better combination to farmers.

## Materials and methods

Field experiment was conducted at IFS Unit, College farm, College of Agriculture, PJTSAU, Rajendranagar during 2021–2022 and 2022–2023 with a view to identify profitable climate smart farming system models under Irrigated Situation of Telangana with suitable crop and animal components. The details of the materials used and the methods adopted during the course of investigation are described in this section.

### Location of the experimental site

**Figure1:**
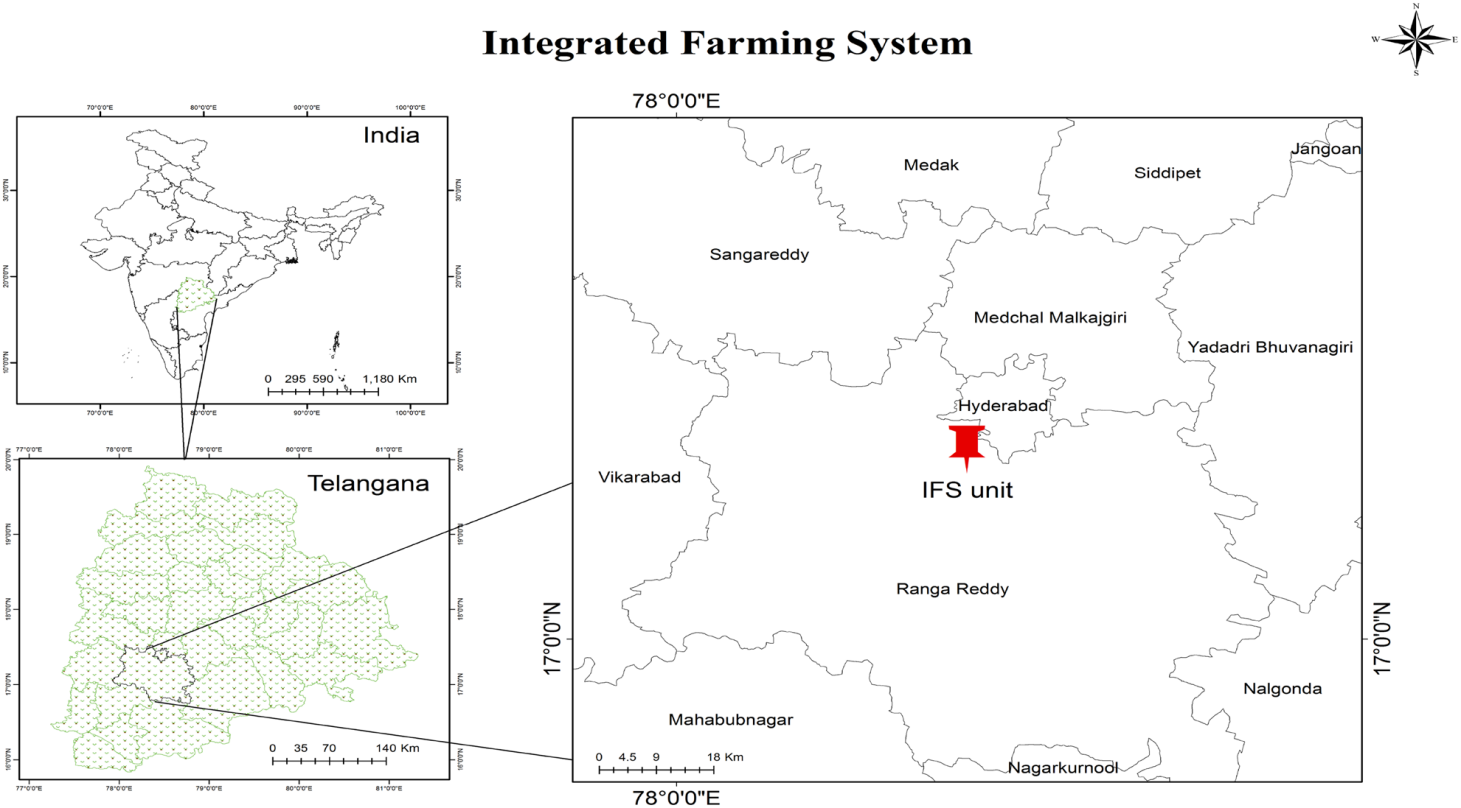
Layout of the experimental field.

The experimental site was situated at an altitude of 527 m above mean sea level (MSL) at 17° 32′ 10.45″ N latitude and 78° 41′ 02.77″ E longitude in Southern Telangana Zone (STZ), India. The experiment was laid out in College of Agriculture, Rajendranagar, Telangana. The layout of the experimental field was depicted in Fig. 1.

### Weather

The meteorological data recorded during the crop growth period of experimentation was taken from the meteorological observatory of Agro Climatic Research Centre (ACRC) located at Agricultural Research Institute, Rajendranagar, Hyderabad. During the study period in 2021–2022, the weekly temperatures ranged from a minimum of 9.6 °C to a maximum of39.2 °C, with respective averages of 20.5 °C and 32.0 °C.

**Figure 2:**
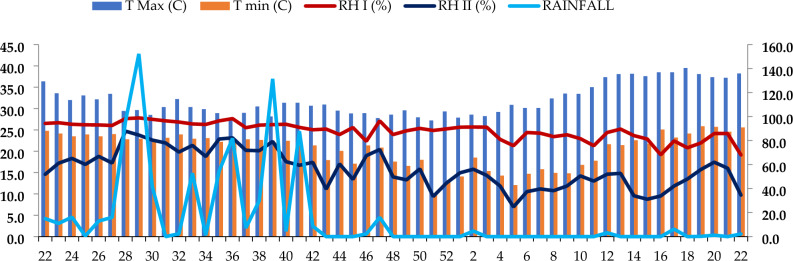
Details of standard week wise meteorological data at Rajendranagar, Telangana during 2021–2022.

The mean weekly morning relative humidity ranged from 67 to 98.9% with an average of 88.1% and evening relative humidity varied from 24.7 to 88.9% with anaverage of 56.3%, respectively. Mean weekly sunshine hours ranged between 1.4 and 10 with anaverage of 6.3. The average annual rainfall was 859.6 mm with 15 rainy days whereas total evaporation was 246.3 mm (Fig. 2).

During the study period in 2022–2023, the weekly temperatures ranged from a minimum of 11.2 °C to a maximum of 39.2 °C, with respective averages of 19.8 °C and 31.9 °C.

**Figure 3:**
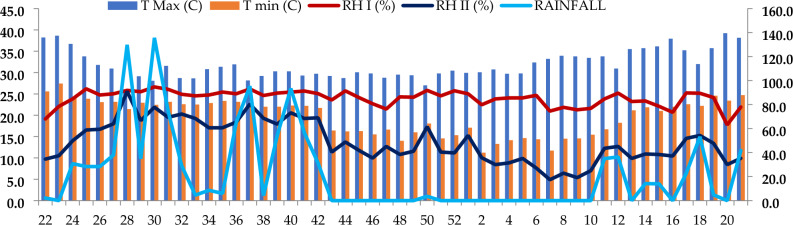
Details of standard week wise meteorological data at Rajendranagar, Telangana during 2022–2023.

The mean weekly morning relative humidity ranged from 63.1 to 94.7%, with an average of 84.9% and evening relative humidity varied from 17.4 to 91.0%, with an average of 48.7%. The mean weekly sunshine hours ranged between 0.3 and 11.0, with an average of 6.7. The average annual rainfall was 1174.4 mm, occurring over 67 rainy days while total evaporation measured was 232.1 mm (Fig. 3).

### Experiment details

Data of this experiment was taken from ongoing research project of AICRP on IFS at Rajendranagar, Hyderabad, Telangana. This experiment consists of different components viz., cropping systems, guava orchard, fodder crops, poultry and sheep. Seven farm models or treatments among which six models (M_2_, M_3_, M_4_, M_5_, M_6_ and M_7_) were developed by combining the various components *i.e.,* cropping systems, fruit crop, fodder crops and livestock components, in different proportions, and compared with rice-groundnut system (M_1_) which is a major farming approach in Telangana. (Table 1). All components were maintained individually from which data was collected and integrated to compare the different IFS models. Acreage of cropping systems varies from model to model which could be seen in Table 2.

**Table 1:** Treatment details and components of various integrated farming system models.

IFS models	C_1_	C_2_	C_3_	C_4_	G	H	N	P	S_1_	S_2_
M_1_	√									
M_2_	√	√	√							
M_3_	√	√		√			√		√	
M_4_	√	√	√	√				√		
M_5_			√		√	√	√			√
M_6_	√		√		√			√		
M_7_	√	√		√		√	√	√		√

**Table 2:** Treatment wise components allocation in 1 acre area.

IFS models	Components	Area
M_1_	Rice–Groundnut	4000 sq m
M_2_	Rice–Groundnut Pigeonpea + Sweetcorn (1:3)—Bajra Bt cotton + Greengram (1:2)—Maize	1000 sq m 1000 sq m 2000 sq m
M_3_	Rice–Groundnut Pigeonpea + Sweetcorn (1:3)—Bajra Pigeonpea + Maize (1:3)—Sunhemp Napier grass Sheep (5 + 1)	1500 sq m 1000 sq m 1000 sq m 500 sq m
M_4_	Rice–Groundnut Pigeonpea + Sweetcorn (1:3)—Bajra Pigeonpea + Maize (1:3)—Sunhemp Bt cotton + Greengram (1:2)—Maize Poultry (100)	1000 sq m 1000 sq m 1000 sq m 1000 sq m
M_5_	Guava Hedge lucerne Napier grass Bt cotton + Greengram (1:2)—Maize Sheep (5 + 1)	2000 sq m 500 sq m 500 sq m 1000 sq m
M_6_	Guava Bt cotton + Greengram (1:2)—Maize Rice–Groundnut Poultry (100)	2000 sq m 1000 sq m 1000 sq m
M_7_	Rice–Groundnut Pigeonpea + Sweetcorn (1:3)—Bajra Pigeonpea + Maize (1:3)—Sunhemp Napier grass Hedge lucerne Sheep (5 + 1) Poultry (100)	1000 sq m 1000 sq m 1000 sq m 500 sq m 500 sq m

+Represents intercrop

−Represents sequence crop.

### Agronomic practices

In integrated farming system, the recommended packages of practices were adopted for achieving higher yield and productivity for all the crops grown under crop and horticulture components given below in Table 3 and the same package of practices were followed in both the years. Land preparation and most of the intercultural operations in all the crops were carried out with the help of tractor drawn implements. All the crops were sown at recommended plant spacing. Transplanting of rice and sowing of remaining crops were carried out manually and plant protection measures were carried out as per recommended schedule. The weed management was carried out using power weeder, wheel hoe and as well as using specific herbicide recommended for a particular crop. All component areas were divided into 3 replications and data was collected from these replications.

**Table 3:** Recommended package of practices of all crops in integrated farming system.

S. No.	Name of the crop	Season	Seed rate unit area^−1^	Spacing	Fertilizer dose ha^−1^	Variety
1	Rice	*Rainy season*	5	20 × 15 cm	120:60:40	RNR 21,278
2	Groundnut	*Winter*	15	22.5 × 10	20:50:30	K 6
3	Pigeonpea	*Rainy season*	0.5	240 × 20	20:50:30	WRG 97
4	Sweetcorn	*Rainy season*	1	60 × 20	200:60:40	Sugar 75
5	Bajra	*Summer*	1	45 × 15	80:40:30	MPMH 21
6	Bt Cotton	*Rainy season*	0.5	90 × 30	150:60:60	Magna (RCH 530 BG II)
7	Greengram	*Rainy season*	2	30 × 10	20:50:30	WGG 42
8	Maize	*Winter*	2	60 × 20	240:80:60	Pioneer 3396
9	Pigeonpea	*Rainy season*	0.5	240 × 20	20:50:30	WRG 97
10	Maize	*Rainy season*	2	60 × 20	240:80:60	Pioneer 3396
11	Sunhemp	*Summer*	4	30 × 10	10:20:0	Local variety
	Fodder crops					
11	Hedge Lucerne	*Perennial*	1 kg	30 cm	40:60:20	RL-88
12	Napier grass	*Perennial*	926cuttings	90 cm × 60 cm	180:60:60	Super napier
	Horticulturalcrops					
13	Guava	*Perennial*		4 × 4 m	100:40:100 2.5 kg Vermicompostplant^−1 ^at the time of planting	Allahabad safeda

#### Manures and fertilizers application

The recommended doses of nutrients (N, P & K) were supplied through urea, SSP and MOP. Entire dose of phosphorous was applied as basal. Nitrogen and Potash were applied as per the schedule of respective crops in both the years.

#### After care

Gap filling and thinning of crops were done on 7th and 15th DAS, respectively based on moisture availability. Hoeing and weeding was done manually and were taken up twice to keep weed free condition. Adequate prophylactic plant protection measures were also carried out to keep crops free from pest and diseases.

#### Irrigation

Irrigation was applied with drip system to every system except for rice-groundnut system and LDPE pipes of 16 mm diameter were used as laterals keeping lateral spacing 60 cm and inline dripper spacing 60 mm with every emitter flow rate of 4 l/h. Flooding method of irrigation was used for rice-groundnut system.

#### Harvesting

Paddywas harvested by cutting the tillers by leaving base of the crop upto 8 cm. Groundnut plants were pulled from the earth, and their pods were methodically stripped away. The entire plants of Pigeonpea and Bajra were carefully uprooted, dried in the sun, and then threshed using sticks. Maize and sweetcorn were harvested by removing the fresh cobs from the plants. Cotton crops were harvested in three successive pickings, and green gram was handpicked from the plants. The phytomass yield of sunhempwas measured at the time of its incorporation into the soil. For each crop, grain and straw yields were meticulously recorded from three separate replications, and the mean yields were subsequently calculated.

### Estimation of rice grain equivalent yield

To compare the productivity of various systems, the yield of each component was converted into RGEY using the formula suggested by^2^. Economic and straw/stover yields were calculated from each replication of unit area and means were calculated which is converted into equivalent yield. Since diversified enterprises were taken in the study, the yield of each enterprise was converted to rice equivalent yield. Studies on economics of production were made by keeping a record on number of labourers engaged, power and input utilized. The prevailing market prices of different commoditiesareused for converting yield into RGEY and for computing the economics. Observations were made on productivity in terms of rice-grain equivalent yield, economics and employment for different farming systems as well as conventional cropping systems.

1 $${\text{Rice grain equivalent yield }} = \frac{{{\text{Yield of a crop}} \times {\text{Market value of a cro}}p}}{{\text{Market value of rice}}}$$

### Sheep

Two units of sheep of Nellore judipi breed are grown (each unit consists of 5 + 1) separately on platform system in partial grazing manner. One unit of sheep were fed napier grass whereas second unit were fed hedge lucerne in addition to napier grass. Every morning sheep are taken for grazing for 4–5 h and they are provided fodder in the shed itself in the evening time. Deworming is done once in 3 months on the adviceof veterinary doctors and they used to visit sheep shed every fortnight for health check-up. Sheep are given serial number and the periodical live weight, growth rate per every 15 days (twice a month) were observed for a period of 24 months (June 2021–May 2023). Sheep manure is collected at the end of year and supplied to the fields.

### Poultry birds

One day old chicks (Aseel breed) are bought from Poultry Research Station, Rajendranagar, Hyderabad. Each batch consisted of 50 birds and two batches were maintained per year. Vaccines are given time to time and everyday chick feed and water are provided as per the requirement. The periodical live weight of poultry birds, increase in live weight and manure production were observed. Once they attain around 1.1 kg weight, they are sold @ ₹300 kg^−1^.

### Economic analysis

Based on the existing market prices of inputs as well as outputs, cost of production and gross returns were calculated. The minimum wage rate is the government fixed wage rate in India and no labour should receive wages below it based on which labour wages are calculated. The cost component in IFS included two types of costs—fixed cost and variable cost. The cost of inputs like seeds, fertilizers, herbicides, pesticides, ploughing, irrigation, labour charges, etc. include variable cost. The one-time initial investment especially in perennial components, construction of animal shed, purchase of animals, establishment of guava, etc. forms the fixed cost. Finally, the net return (gross return–total cost) and benefit cost ratio (Gross returns/cost of production) were calculated.23

### System economic efficiency

System economic efficiency (SEE) was calculated to know net returns obtained per day. SEE was estimated based on the net returns obtained in an IFS model in a year and divided by 365. It was calculated by using the formula suggested by^2^.4

### Sustainable yield index

Sustainable Yield Index (SYI) was calculated by using the formula suggested by^13^.

5 $$SYI=\frac{Ymean - S. D.Y }{Ymax}$$

where, Y_mean_ is mean yield obtained from any IFS model, S.D.Y is standard deviation of mean yields of all IFS models, Y_max_ is maximum yield obtained from any model.

### Sustainable value index

Sustainable Value Index (SVI) was calculated by using the formula suggested by^14^

6 $$SVI=\frac{Nmean - S. D.N }{Nmax}$$

where, N_mean_ is mean net returns obtained from any IFS model, S.D.N is standard deviation of mean net returns of all IFS models, N_max_ is maximum net returns obtained from any model.

### Water footprints

Water footprint is amount of water required to produce a kg of produce which is measured to identify the water efficient models. The amount of water used by each crop in the cropping system and livestock is added to obtain the total water usage (in l) in each model. The total water usage of each model is divided by rice grain equivalent yield to obtain water foot prints and is expressed in l kg^−1^ as suggested by^15^.

### Statistical analysis

The data were analyzed statistically by applying one way “Analysis of Variance” (ANOVA) technique of randomized block design^16^. The significance of different sources of variations was tested by error mean square of Fisher Snedecor’s ‘F’ test at probability level 0.05. Standard error of mean (SEm ±) and critical difference (CD) at 5% level of significance were worked out for each character and provided in the tables of the results to compare the difference between the treatment means.

### Approval for plant and animal experiments

All the methods followed in the study comply with the Professor Jayashankar Telangana State Agricultural University (PJTSAU), Hyderabad, Telangana, India and IIFSR-Modipuram, India guidelines. All the authors abide by the IUCN Policy Statement on Research Involving Species at Risk of Extinction and the Convention on the Trade in Endangered Species of Wild Fauna and Flora. All the experiment protocols in the experiment were approved by Professor Jayashankar Telangana State Agricultural University (PJTSAU), Hyderabad, Telangana, India and all the methods were carried out in accordance with PJTSAU and ICAR guidelines and regulations. All methods are reported in accordance with ARRIVE guidelines.

## Results

### Yield

Rice crop has recorded a grain and straw yield of 519.5 and 602.5 kg 1000 sq m 1000 sq m^−1^, respectively in 2021–2022 whereas it has recorded grain and straw yield of 530 and 671 kg 1000 sq m 1000 sq m^−1^, respectively in 2022–2023. Mean grain and straw yield of rice crop were 525 and 637 kg 1000 sq m 1000 sq m^−1^, respectively. In the year 2021–2022, podandhaulmyields of groundnut crop were 209.5 and 345.5 kg 1000 sq m 1000 sq m^−1^, respectively whereas podandhaulmyields in 2022–2023 were 215 and 363 kg 1000 sq m 1000 sq m^−1^, respectively. Mean podandhaulmyields of groundnut crop were 212 and 354 kg 1000 sq m 1000 sq m^−1^, respectively (Table 4).

**Table 4:** Productivity of various crops in cropping systems of integrated farming system.

Components	Season	Area (sq m)	Yield(kg)					
			2021–2022		2022–2023		Mean	
			Grain/kapas/fruit yield	Straw/stover yield	Grain/kapas/fruit yield	Straw/stover yield	Grain/kapas/fruit yield	Straw/stover yield
Cropping system-I								
Rice	*Rainy season*	1000	519.5	602.5	530	671	525	637
Groundnut	*Winter*	1000	209.5	345.5	215	363	212	354
Cropping system-II								
Pigeonpea + Sweetcorn (1:3)	*Rainy season*	1000	55 + 956	204 + 1147	62 + 1173	243 + 1408	58.5 + 1064.5	223.5 + 1277.5
Bajra	*Winter*	1000	254	457	261	477	257.5	467
Cropping system-III								
Bt Cotton + Greengram (1:2)	*Rainy season*	1000	154 + 47	363 + 102	205 + 47	461 + 110	179.5 + 47	412 + 106
Maize	*Winter*	1000	540	670	580	725	560	697.5
Cropping system-IV								
Pigeonpea + Maize (1:3)	*Rainy season*	1000	61 + 563	219 + 721	57 + 586	233 + 738	59 + 574.5	226 + 729.5
Sunhemp	*Winter*	1000		1785		1809		1797
Horticulture								
Guava Orchard	Perennial	2000	342		378		360	
Forage crops								
Hedge Lucerne	Perennial	500		4304		5248		4776
Napier grass	Perennial	500		12,821		13,089		12,955

Pigeonpea has recorded a grain and stoveryield of 55 and 204 kg 1000 sq m 1000 sq m^−1^, respectively in 2021–2022 whereas it has recorded grain and stoveryield of 62 and 243 kg 1000 sq m 1000 sq m^−1^, respectively in 2022–2023. Mean grain and stoveryield of pigeonpea crop were 58.5 and 223.5 kg 1000 sq m^−1^, respectively. In the year 2021–2022, grain and straw yield of sweetcorn crop were 956 and 1147 kg 1000 sq m^−1^, respectively whereas grain and straw yield in 2022–2023 were 1173 and 1408 kg 1000 sq m^−1^, respectively. Mean grain and straw yield of sweetcorn crop were 1064.5 and 1277.5 kg 1000 sq m^−1^, respectively. Bajra crop has recorded a grain and straw yield of 254 and 457 kg 1000 sq m^−1^, respectively in 2021–2022 whereas it has recorded grain and straw yield of 261 and 477 kg 1000 sq m^−1^, respectively in 2022–2023. Mean grain and straw yield of bajra crop were 257.5 and 467 kg 1000 sq m^−1^, respectively (Table 4).

Bt cotton has recorded a seed cottonandstalkyields of 154 and 363 kg 1000 sq m^−1^, respectively in 2021–2022 whereas it has recorded seed cottonandstalkyields of 205 and 461 kg 1000 sq m^−1^, respectively in 2022–2023. Mean seed cottonandstalkyields of Bt cotton were 179.5 and 412 kg 1000 sq m^−1^, respectively. In the year 2021–2022, grain and stoveryields of greengram crop were 47 and 102 kg 1000 sq m^−1^, respectively whereas grain and stoveryields in 2022–2023 were 47 and 110 kg 1000 sq m^−1^, respectively. Mean grain and stoveryields of greengram crop were 47 and 106 kg 1000 sq m^−1^, respectively. Maize crop has recorded a grain and straw yield of 540 and 670 kg 1000 sq m^−1^, respectively in 2021–2022 whereas it has recorded grain and straw yield of 580 and 725 kg 1000 sq m^−1^, respectively in 2022–2023. Mean grain and straw yield of maize crop were 560 and 697.5 kg 1000 sq m^−1^, respectively (Table 4).

Pigeonpea has recorded a grain and stoveryields of 61 and 219 kg 1000 sq m^−1^, respectively in 2021–2022 whereas it has recorded grain and stoveryields of 57 and 233 kg 1000 sq m^−1^, respectively in 2022–2023. Mean grain and stoveryields of pigeonpea crop were 59 and 226 kg 1000 sq m^−1^, respectively. In the year 2021–2022, grain and straw yield of maize crop were 563 and 721 kg 1000 sq m^−1^, respectively whereas grain and straw yield in 2022–2023 were 586 and 738 kg 1000 sq m^−1^, respectively. Mean grain and straw yield of sweetcorn crop were 574.5 and 729.5 kg 1000 sq m^−1^, respectively. Sunhemp crop has recorded a fodder yield of 1785 and 1809 kg 1000 sq m^−1^ in both the years, respectively. Mean fodder yield of sunhemp was 1797 kg 1000 sq m^−1^(Table 4).

Guava orchard has recorded fruit yield of 342 and 378 kg 2000 sq m^−1^ in both the years, respectively and mean yield of 360 kg 2000 sq m^−1^ was obtained. Hedge lucerne has recorded a fodder yield of 4304 and 5248 kg 500 sq m^−1^ in both the years, respectively and mean fodder yield of 4776 kg 500 sq m^−1^ was obtained. Napier grass has recorded a fodder yield of 12,821 and 13,089 kg 500 sq m^−1^ in both the years, respectively and mean fodder yield of 12,955 kg 500 sq m^−1^ was obtained (Table 4).

### Sheep

**Figure 4:**
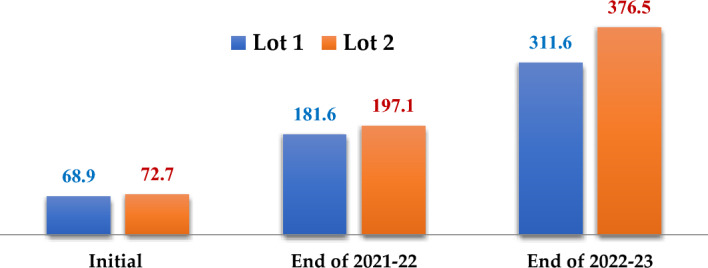
Weight gain in sheep lot I and II over the 2 years.

Two sheep lots were maintained which differs in feed and each lot consists of 5 + 1 sheep (5 Female + 1 Male). Lot I mainly was mainly fed napier grass, silage & dry fodder and lot II was fed hedge lucerne in addition to feed of lot I. Initial weights of sheep lot I and lot II were 68.9 and 72.7 kg, respectively whereas final weights were 181.6 and 197.1 kg, respectively at the end of agricultural year 2021–2022 (Fig. 4).

Mean growth rates of lot I and lot II were 4.54 and 5.18 kg fortnight^−1^. Final weights of lot I and lot II were 330.6 and 376.5 kg, respectively at the end of agricultural year 2022–2023 (Table 5). Mean growth rates of lot I and lot II were 6.21 and 7.48 kg fortnight^−1^. Ramana et al.^17^ found that lambs and kids grazed on silvipasture gained 54.8 and 36.8 ghead^−1^/day^−1^, respectively whereas on natural grassland showed 41.2 and 26.4 ghead^−1^ day^−1^, respectively. Lot I has increased from 5 + 1 to 9 + 7 and Lot I has increased from 5 + 1 to 11 + 8 at the end of agricultural year 2022–2023. Higher growth rate of lot II compared to Lot I might be due to better feed which has resulted in good health as well as production of more number of kids which have contributed to overall weight of the lot. Growth rate in the 2nd year compared to 1st year is might be due to production of more number of kids which have contributed to overall weight of the lot. Manure production of Lot I and lot II were 416 and 432 kg year^−1^, respectively in the year 2021–2022 whereas manure production of Lot I and lot II were 1081 and 1286 kg year^−1^, respectively in the year 2022–2023. Much variation was observed in manure production between two lots in the 2^nd^ year which is mainly because of difference in the number of sheep.

**Table 5:** Sheep production during 2021–2022 and 2022–2023.

Breed (Nellore Judipi)	2021–2022	2022–2023
Lot I		
Population	5 + 1	9 + 7
Initial weight (kg)	68.9	181.6
Final weight (kg)	181.6	311.6
Increase inweight (kg)	112.70	130.0
Manure(kg)	416	1081
Lot II		
Population	5 + 1	11 + 8
Initial weight (kg)	72.7	197.1
Final weight (kg)	197.1	376.5
Increase in weight (kg)	124.4	179.4
Manure(kg)	432	1286

### Poultry

Two batches of Aseel poultry birds per year were maintained and each batch consisted of 50 birds. Total four batches were maintained in 2 years of research work. One day old chicks were bought and sold after attaining weight around 1.2 kgs. In the year 2021–2022, Initial weights of batch I and II were 1.27 and 1.26 kg, respectively and weights at the time of sale were 52 and 50 kgs, respectively. 6 birds were died in the batch I whereas 8 birds were died in the batch II due to fatty liver disease, sudden changes in the temperatures. Batch I has produced manure of 46 kg and batch II has produced 44 kg (Table 6).

**Table 6:** Poultry yield in both the years.

Breed (Aseel)	Yield	
	2021–2022	2022–2023
	**Batch I (50 birds)**	**Batch III (50 birds)**
Initial weight (kg)	1.27	1.30
Weight at the time of sale (kg)	52	55
Manure(kg)	46	49
	**Batch II (50 birds)**	**Batch IV (50 birds)**
Initial weight (kg)	1.26	1.29
Weight at the time of sale (kg)	50	53
Manure(kg)	44	47

In the year 2022–2023, Initial weights of batch I and II were 1.30 and 1.29 kgs, respectively and weights at the time of sale were 55 and 53 kgs, respectively. 4 birds were died in the batch I whereas 6 birds were died in the batch II. Batch I has produced manure of 49 kg and batch II has produced 47 kg. Maize grown in our field was mainly used as feed for poultry. Total manure produced in the 1st year was 90 kg and in the 2nd year, 96 kg was produced. Mortality rate in the 1st year was 14% where it is reduced to 10% in the 2nd year which is mainly due to better feed as well as management practices.

### Productivity of various components (In terms of RGEY)

Rice has obtained 550.5 and 563 RGEY kg 1000 sq m^−1^ in 2021–2022 and 2022–2023, respectively whereas mean RGEY is 556.75 kg 1000 sq m^−1^. Groundnut has obtained 658.5 and 671 RGEY kg 1000 sq m^−1^ in 2021–2022 and 2022–2023, respectively whereas mean RGEY is 664.75 kg 1000 sq m^−1^. Overall rice-groundnut system has achieved RGEY of 1209 and 1234 kg 1000 sq m^−1^ in 2021–2022 and 2022–2023, respectively whereas mean RGEY obtained is 1221.5 kg 1000 sq m^−1^ (Table 7).

**Table 7:** Rice equivalent yields of different components of integrated farming system.

Components	Season	Area (sq m)	Rice grain equivalent yields (kg 1000 sq m^−1^)		
			2021–2022	2022–2023	Mean
CroppingSystem-I					
Rice	*Rainy season*	1000	550.5	563	556.75
Groundnut	*Winter*	1000	658.5	671	664.75
Rice- Groundnut			1209	1234	1221.5
CroppingSystem-II					
Pigeonpea + Sweetcorn (1:3)	*Rainy season*	1000	714	800	757
Bajra	*Winter*	1000	319	348	333.5
Pigeonpea + Sweetcorn (1:3)- Bajra			1033	1148	1090.5
CroppingSystem-III					
Bt Cotton + Greengram (1:2)	*Rainy season*	1000	647	848	747.5
Maize	*Winter*	1000	556	593	574.5
Bt Cotton + Greengram (1:2)- Maize			1203	1441	1322
CroppingSystem-IV					
Pigeonpea + Maize (1:3)	*Rainy season*	1000	781	794	787.5
Sunhemp	*Winter*	1000	184	177	180.5
Pigeonpea + Maize (1:3)- Sunhemp			965	971	968
Horticulture					
Guava Orchard	Perennial	2000	529	556	542.5
Forage crops					
Hedge Lucerne	Perennial	500	666	772	719
HybridNapier	Perennial	500	1982.5	1925	1953.75
Livestock					
Poultry		100 birds	1579	1588	1584
Sheep lot I		5 + 1	1859	2052	1956
Sheep lot II		5 + 1	2052	2830	2441

Pigeonpea + Sweetcorn system has obtained 714 and 800 RGEY kg 1000 sq m^−1^ in 2021–2022 and 2022–2023, respectively whereas mean RGEY is 757 kg 1000 sq m^−1^. Bajra has obtained 319 and 348 RGEY kg 1000 sq m^−1^ in 2021–2022 and 2022–2023, respectively whereas mean RGEY is 333.5 kg 1000 sq m^−1^. Overall, Pigeonpea + Sweetcorn (1:3)—Bajra system has achieved RGEY of 1033 and 1148 kg 1000 sq m^−1^ in 2021–2022 and 2022–2023, respectively whereas mean RGEY obtained is 1090.5 kg 1000 sq m^−1^ (Table 7).

Bt cotton + Greengram system has obtained 647 and 848 RGEY kg 1000 sq m^−1^ in 2021–2022 and 2022–2023, respectively whereas mean RGEY is 747.5 kg 1000 sq m^−1^. Maize has obtained 556 and 593 RGEY kg 1000 sq m^−1^ in 2021–2022 and 2022–2023, respectively whereas mean RGEY is 574.5 kg 1000 sq m^−1^. Overall Bt Cotton + Greengram (1:2)—Maize system has achieved RGEY of 1203 and 1441 kg 1000 sq m^−1^ in 2021–2022 and 2022–2023, respectively whereas mean RGEY obtained is 1322 kg 1000 sq m^−1^ (Table 7).

Pigeonpea + Maize system has obtained 781 and 794 RGEY kg 1000 sq m^−1^ in 2021–2022 and 2022–2023, respectively whereas mean RGEY is 787.5 kg 1000 sq m^−1^. Sunhemp has obtained 184 and 177 RGEY kg 1000 sq m^−1^ in 2021–2022 and 2022–2023, respectively whereas mean RGEY is 180.5 kg 1000 sq m^−1^. Overall, Pigeonpea + Maize (1:3)—Sunhemp system has achieved RGEY of 965 and 971 kg 1000 sq m^−1^ in 2021–2022 and 2022–2023, respectively whereas mean RGEY obtained is 968 kg 1000 sq m^−1^ (Table 7).

Guava orchard has obtained 529 and 556 RGEY kg 2000 sq m^−1^ in 2021–2022 and 2022–2023, respectively whereas mean RGEY is 542.5 kg 2000 sq m^−1^. Hedge lucerne has obtained 666 and 772 RGEY kg 500 sq m^−1^ in 2021–2022 and 2022–2023, respectively whereas mean RGEY is 719 kg 500 sq m^−1^. Napier grass system has obtained 1982.5 and 1925 RGEY kg 500 sq m^−1^ in 2021–2022 and 2022–2023, respectively whereas mean RGEY is 1953.75 kg 500 sq m^−1^ (Table 7).

Poultry unit has obtained 1579 and 1588 RGEY kg unit^−1^ in 2021–2022 and 2022–2023, respectively whereas mean RGEY is 1584 kg unit^−1^. Sheep unit I has obtained 1859 and 2052 RGEY kg unit^−1^ in 2021–2022 and 2022–2023, respectively whereas mean RGEY is 1956 kg unit^−1^. Sheep unit II has obtained 2052 and 2830 RGEY kg unit^−1^ in 2021–2022 and 2022–2023, respectively whereas mean RGEY is 2441 kg unit^−1^ (Table 7).

### System productivity (RGEY kg acre^−1^)

Among all the models, M_7_ is superior to rest of the models which has obtained the higher system productivity followed by M3 (Table 8). Models M_6_ and M_1_ (Traditional rice-groundnut system) have obtained lower system productivity comared to remaining models which indicates that integration of suitable components results in higher productivity and vice versa.

**Table 8:** Component and system productivity in integrated farming systems.

IFSmodels	System Productivity (RGEY kg acre^−1^)		
	2021–2022	2022–2023	Mean
**M**_**1**_: C_1_	4836d	4936d	4886e
**M**_**2**_: C_1_ + C_2_ + C_3_	4648d	5264d	4956e
**M**_**3**_: C_1_ + C_2_ + C_4_ + N + S_1_	7653b	7948b	7800b
**M**_**4**_: C_1_ + C_2_ + C_3_ + C_4_ + P	5987c	6382c	6185d
**M**_**5**_: G + H + N + C_3_ + S_2_	6439c	7524b	6981c
**M**_**6**_: G + C_1_ + C_3_ + P	4520d	4819d	4670e
**M**_**7**_: C_1_ + C_2_ + C_4_ + H + N + S_2_ + P	9493a	10468a	9981a

C_1_:Rice–Groundnut; C_2_:Pigeonpea + Sweetcorn (1:3)—Bajra; C_3_: Bt cotton + Greengram (1:2)—Maize; C_4_: Pigeonpea + Maize (1:3)—Sunhemp; G-Guava; N: Napier grass; H-Hedge lucerne; P: Poultry; S_1_: Sheep lot I; S_2_: Sheep lot II.

### Economics of various components

Rice–Groundnut system has obtained gross & net returns of ₹23,460 and ₹12,730, respectively with cost of production of ₹10,730 and B:C ratio of 2.19 in the 2021–2022 and gross & net returns of ₹25,173 and ₹14,028, respectively with cost of production of ₹11,145 and B:C ratio of 2.26 in the 2022–2023. Mean gross and net returns were ₹24,317 and ₹13,379, respectively with cost of production of ₹ 10,938 and B:C ratio of 2.22 (Table 9).

**Table 9:** Economicsof various componentsin integrated farming systems.

Cropcomponents	Season	Area (sq m)	2021–2022				2022–2023				Mean			
			Cost ofcultivation	Grossreturns	Netreturns	B:Cratio	Cost ofcultivation	Grossreturns	Netreturns	B:Cratio	Cost ofcultivation	Grossreturns	Netreturns	B:Cratio
CroppingSystem-I														
Rice	*Rainy season*	1000	5322	10,681	5359	2.00	5575	11,485	5910	2.06	5449	11,083	5634	2.03
Groundnut	*Winter*	1000	5408	12,779	7371	2.36	5570	13,688	8118	2.46	5489	13,234	7745	2.41
Rice-Groundnut			10,730	23,460	12,730	2.19	11,145	25,173	14,028	2.26	10,938	24,317	13,379	2.22
CroppingSystem-II														
Pigeonpea + Sweetcorn (1:3)	*Rainy season*	1000	7320	13,840	6520	1.89	7560	16,320	8760	2.16	7440	15,080	7640	2.03
Bajra	*Winter*	1000	3460	6180	2720	1.79	3550	7099	3549	2.00	3505	6640	3135	1.89
Pigeonpea + Sweetcorn(1:3)—Bajra			10,780	20,020	9240	1.86	11,110	23,419	12,309	2.11	10,945	21,720	10,775	1.98
CroppingSystem-III														
Bt Cotton + Greengram (1:2)	*Rainy season*	1000	6757	13,406	6649	1.98	7063	17,299	10,236	2.45	6910	15,353	8943	2.22
Maize	*Winter*	1000	4961	9933	4972	2.00	5108	12,097	6989	2.37	5035	11,015	5980	2.19
Bt Cotton + Greengram (1:2)—Maize			11,718	23,339	11,621	2.00	12,171	29,396	17,225	2.42	11,945	26,368	14,423	2.21
CroppingSystem-IV														
Pigeonpea + Maize (1:3)	*Rainy season*	1000	5805	15,147	9342	2.61	5865	16,198	10,333	2.76	5835	15,673	9838	2.69
Sunhemp	*Winter*	1000	1675	3573	1898	2.13	1710	3611	1901	2.11	1693	3592	1899	2.12
Pigeonpea + Maize (1:3)—Sunhemp			7480	18,720	11,240	2.50	7575	19,809	12,234	2.62	7528	19,265	11,737	2.56
Horticulture														
Guava Orchard	Perennial	2000	4140	10,260	6120	2.48	4530	11,343	6813	2.50	4335	10,802	6467	2.49
Forage crops														
Hedge Lucerne	Perennial	500	1935	12,920	10,985	6.68	2475	15,752	13,277	6.36	2205	14,336	12,131	6.50
Hybrid Napier	Perennial	500	2815	38,460	35,645	13.66	3360	39,270	35,910	11.69	3088	38,865	35,777	12.59
Livestock unit														
Poultry unit		100 birds	15,015	30,626	15,611	2.04	15,268	32,395	17,127	2.12	15,142	31,511	16,369	2.08
Sheep lot I			41,308	58,162	16,854	1.41	22,944	41,870	18,926	1.82	32,126	50,016	17,890	1.56
Sheep lot II			43,418	63,123	19,705	1.45	27,866	57,730	29,864	2.07	35,642	60,427	24,785	1.70

Pigeonpea + Sweetcorn (1:3)—Bajra system has obtained gross & net returns of ₹20,020 and ₹9240, respectively with cost of production of ₹10,780 and B:C ratio of 1.86 in the 2021–2022 and gross & net returns of ₹23,419 and ₹12,309, respectively with cost of production of ₹11,110 and B:C ratio of 2.11 in the 2022–2023. Mean gross and net returns were ₹21,720 and ₹10,775, respectively with cost of production of ₹ 10,945 and B:C ratio of 1.98 (Table 9).

Bt cotton + Greengram (1:2)—Maize system has obtained gross & net returns of ₹23,339&₹11,621, respectively with cost of production of ₹11,718 and B:C ratio of 2.00 in the 2021–2022 and gross & net returns of ₹29,396 and ₹17,225, respectively with cost of production of ₹12,171 and B:C ratio of 2.42 in the 2022–2023. Mean gross and net returns were ₹26,368 and ₹14,423, respectively with cost of production of ₹ 11,945 and B:C ratio of 2.21 (Table 9).

Pigeonpea + Maize (1:3)—Sunhemp system has obtained gross & net returns of ₹18,720 and ₹ 11,240, respectively with cost of production of ₹7480 and B:C ratio of 2.50 in the 2021–2022 and gross & net returns of ₹19,809 and ₹12,234, respectively with cost of production of ₹7575 and B:C ratio of 2.62 in the 2022–2023. Mean gross and net returns were ₹19,265 and ₹11,737, respectively with cost of production of ₹ 7528 and B:C ratio of 2.56 (Table 9).

Guava orchard has obtained gross & net returns of ₹10,260 &₹ 6120, respectively with cost of production of ₹4140 and B:C ratio of 2.48 in the 2021–2022 and gross & net returns of ₹11,343 and ₹6813, respectively with cost of production of ₹4530 and B:C ratio of 2.50 in the 2022–2023. Mean gross and net returns were ₹10,802 and ₹6467, respectively with cost of production of ₹4335 and B:C ratio of 2.49. Hedge lucerne has obtained gross & net returns of ₹12,920 and ₹10,985, respectively with cost of production of ₹1935 and B:C ratio of 6.68 in the 2021–2022 and gross and net returns of ₹15,752 and ₹13,277, respectively with cost of production of ₹2475 and B:C ratio of 6.36 in the 2022–2023. Mean gross and net returns were ₹14,336 and ₹12,131, respectively with cost of production of ₹2205 and B:C ratio of 6.50. Napier grasshas obtained gross and net returns of ₹38,460 and ₹35,645, respectively with cost of production of ₹2815 and B:C ratio of 13.66 in the 2021–2022 and gross and net returns of ₹39,270 and ₹35,910, respectively with cost of production of ₹3360 and B:C ratio of 11.69 in the 2022–2023. Mean gross and net returns were ₹38,865 and ₹35,777, respectively with cost of production of ₹ 3088 and B:C ratio of 12.59 (Table 9).

Poultry unit has obtained gross & net returns of ₹30,626 &₹15,611, respectively with cost of production of ₹15,015 and B:C ratio of 2.04 in the 2021–2022 and gross & net returns of ₹32,395 &₹17,127, respectively with cost of production of ₹15,268 and B:C ratio of 2.12 in the 2022–2023. Mean gross and net returns were ₹31,511 &₹15,142, respectively with cost of production of ₹16,369 and B:C ratio of 2.08 (Table 9).

Sheep unit I has obtained gross & net returns of ₹58,162 &₹16,854, respectively with cost of production of ₹41,308 and B:C ratio of 1.41 in the 2021–2022 and gross & net returns of ₹41,870 &₹18,926, respectively with cost of production of ₹22,944 and B:C ratio of 1.82 in the 2022–2023. Mean gross and net returns were ₹50,016 &₹17,890, respectively with cost of production of ₹32,126 and B:C ratio of 1.56 (Table 9).

Sheep unit II has obtained gross & net returns of ₹63,123 &₹19,705, respectively with cost of production of ₹43,418 and B:C ratio of 1.45 in the 2021–2022 and gross & net returns of ₹57,730 &₹29,864, respectively with cost of production of ₹27,866 and B:C ratio of 2.07 in the 2022–2023. Mean gross and net returns were ₹60,427 &₹24,785 with cost of production of ₹35,642 and B:C ratio of 1.70 (Table 9).

### Economics of different integrated farming system models

Among the different integrated farming system models, M_7_ had recorded higher gross & net returns (Table 10) followed by M_3_. Model M_5_ had recorded higher B:C in both the years compared to other models because of lower cost of production and higher returns. Conventional system (M_1_) had recorded lower gross & net returns.

**Table 10:** Economics of different integrated farming system models.

IFS models	2021–2022				2022–2023				Mean			
	Cost of production	Gross Returns	Net Returns	B:C Ratio	Cost of production	Gross Returns	Net Returns	B:C ratio	Cost of production	Gross Returns	Net Returns	B:C ratio
	(₹ acre^−1^)				(₹ acre^−1^)				(₹ acre^−1^)			
**M**_**1**_: C_1_	42920e	93840e	50920d	2.19*	44580d	100692d	56112d	2.26c	43,750	97266e	53516d	2.22*
**M**_**2**_: C_1_ + C_2_ + C_3_	44946e	90158e	45212d	2.01*	46597d	107384d	60787d	2.30c	45,772	98771e	53000d	2.16*
**M**_**3**_: C_1_ + C_2_ + C_4_ + N + S_1_	78478b	170552b	92074b	2.17*	61707b	162128b	100421b	2.63b	70,092	166340b	96247b	2.37*
**M**_**4**_: C_1_ + C_2_ + C_3_ + C_4_ + P	55723d	116165d	60442c	2.08*	57269bc	130192c	72923c	2.27c	56,496	123179d	66683c	2.18*
**M**_**5**_: G + H + N + C_3_ + S_2_	64026c	148102c	84076b	2.31*	50402 cd	153491b	103089b	3.05a	57,214	150797c	93583b	2.64*
**M**_**6**_: G + C_1_ + C_3_ + P	41603e	87685e	46082d	2.11*	43114d	98307d	55193d	2.28c	42,359	92996e	50638d	2.20*
**M**_**7**_: C_1_ + C_2_ + C_4_ + H + N + S_2_ + P	92173a	207329a	115156a	2.25*	78799a	213548a	134749a	2.71b	85,486	210439a	124953a	2.46*

C_1_:Rice–Groundnut; C_2_:Pigeonpea + Sweetcorn (1:3)—Bajra; C_3_: Bt cotton + Greengram (1:2)—Maize; C_4_: Pigeonpea + Maize (1:3)—Sunhemp; G-Guava; N: Napier grass; H-Hedge lucerne; P: Poultry; S_1_: Sheep lot I; S_2_: Sheep lot II.

*Non significant.

### System economic efficiency (₹ day^−1^) and Sustainability index

**Figure 5:**
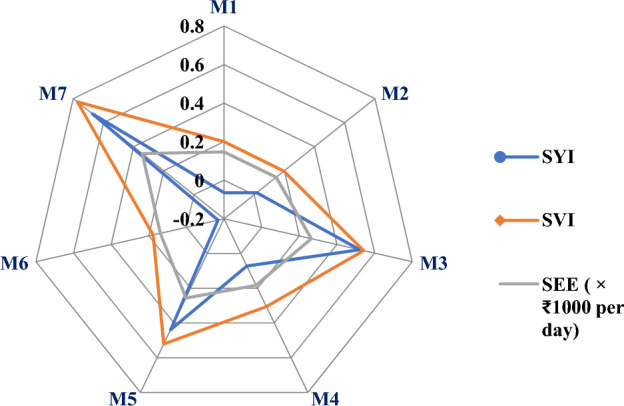
Sustainable yield (SYI), value index (SVI) and system economic efficiencyof different integrated farming system models.

Among all the models, M_7_ had obtained highest mean system economic efficiency followed by model M_3_ and M_5_. Model M_6_ had recorded lowest mean system economic efficiency followed by M_2_ and M_1_ (Fig. 5). Among all the models, M_7_ has recorded highest sustainable yield index and value index followed by M_3_ and M_5_ (Table 11). Models M_1_ and M_6_ have obtained negative sustainable yield index which might be due to higher standard deviation of all the seven modules as compared to yield of these two systems. Models M_6_ and M_2_ have obtained low sustainable value index (Fig. 5) which might be due to low net returns of these models.

**Table 11:** Water footprints of different integrated farming system models.

IFS Models	RGEY (kg acre^−1^)			Water usage (× 10^3^ lit acre^−1^)			Water footprints (litre kg^−1^)		
	2021–2022	2022–2023	Mean	2021–2022	2022–2023	Mean	2021–2022	2022–2023	Mean
**M**_**1**_: C_1_	4836d	4936d	4886e	3800d	3400d	3600d	785.8e	688.8e	737.3d
**M**_**2**_: C_1_ + C_2_ + C_3_	4648d	5264d	4956e	2600a	2566abc	2583a	559.4c	487.5c	523.4 cd
**M**_**3**_: C_1_ + C_2_ + C_4_ + N + S_1_	7653b	7948b	7800b	3178bc	2637abc	2908bc	415.3b	331.8b	373.5b
**M**_**4**_: C_1_ + C_2_ + C_3_ + C_4_ + P	5987c	6382c	6185d	2800ab	2436ab	2618ab	467.7b	381.7b	424.7bc
**M**_**5**_: G + H + N + C_3_ + S_2_	6439c	7524b	6982c	2776ab	2534abc	2655ab	431.1b	336.8b	384.0b
**M**_**6**_: G + C_1_ + C_3_ + P	4520d	4819d	4670e	3130b	2832c	2981c	692.5d	587.7d	640.1d
**M**_**7**_: C_1_ + C_2_ + C_4_ + H + N + S_2_ + P	9493a	10468a	9981a	2804ab	2330a	2567a	295.4a	222.6a	259.0a

C_1_: Rice–Groundnut; C_2_: Pigeonpea + Sweetcorn (1:3)—Bajra; C_3_: Bt cotton + Greengram (1:2)—Maize; C_4_: Pigeonpea + Maize (1:3)—Sunhemp; G-Guava; N: Napier grass; H-Hedge lucerne; P: Poultry; S_1_: Sheep lot I; S_2_: Sheep lot II.

### Water footprints of different integrated farming system models

Water footprint is amount of water required to produce a kg of produce which is measured to identify the water efficient models. Among all the integrated farming system models, model M_7_ had recorded lowest water footprints in both the years, respectively followed by M_3_ and M_5_ (Table 11). Models M_1_ had obtained highest water footprints in both the years, respectively because of high water requirement of rice crop. Model M_6_ also recorded the higher water foot prints in both the years, respectively because of low RGEY.

## Discussion

### Productivity

Bt Cotton + Greengram (1:2)—Maize system has recorded higher RGEY among all the cropping systems which might be due to higher yield of maize and higher yield as well as price of Bt cotton. Among all the components, sheep units have achieved higher RGEY followed by napier grass because of higher growth rate and price of sheep. Higher productivity and fast growth rate have contributed to higher RGEY of napier grass.

M_7_ has obtained the higher productivity mainly because of having multiple components, higher production of napier grass and higher production as well as pricing of sheep meat. Growing different crops with different requirements kept the pest and weed population in check and enhanced the overall productivity. These results in agreement with^18,19^ who found that livestock components would enhance the economic condition of marginal farmers owing to higher price and faster growth rate. M_7_ has obtained around 104% higher system productivity as compared to traditional system because of inclusion of profitable enterprises like Bt cotton + Greengram, Pigeonpea + Sweetcorn, sheep and napier grass. Livestock components enhances the overall system productivity as well as profitability compared to traditional cropping systems^13^. Integration of livestock with cropping systems could solve the problems of small and marginal farmers who occupy majority of farm holdings in India.

### Economics

Among the cropping systems, Bt cotton + Greengram (1:2)—Maize system has obtained higher gross as well as net returns followed by rice-groundnut system. This might be due to higher yield and price of Bt cotton and higher yield of maize. Pigeonpea + Maize (1:3)—Sunhemp system has obtained higher B:C ratio mainly because of lower cost of cultivation (Table 9).

Among all the components, sheep lot II, sheep lotI and napier grass has obtained gross and net returns which might be due to higher meat demand and better growth rate. Higher meat production in sheep lot II compared to lot I is might be due to better feed which has resulted in better growth rate.^20^ reported that inclusion of small ruminants like sheep/goat in farming systems improves the income of farmers and saves them from crop failure. Napier grass has higher fodder production capacity at faster growth rate and higher demand which has resulted in higher gross as well as net returns and superior B:C ratio due to low cost of production which is mainly attributed to faster re-growth and low pest incidence.

**Figure 6:**
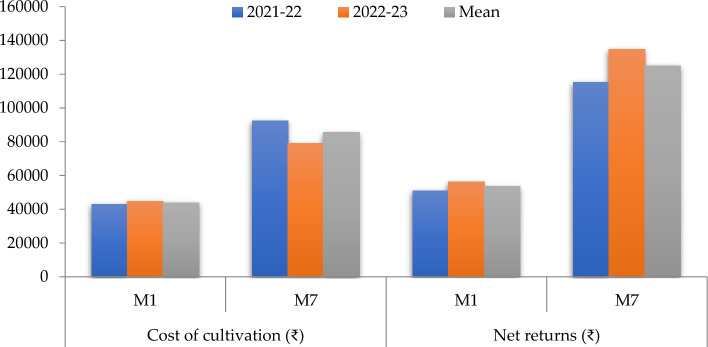
Comparison between economics of M_1_ (existing) and M_7_ (recommended) models.

Higher income is obtained in M_7_, M_5_ and M_3_ models is mainly because of having multiple enterprises, which have complementary interactions between them and produces income throughout the year unlike conventional systems. Sheep and napier grass have contributed much to the income because of their demand and year round production. These results are in agreement with^15,21^ who found that higher returns in integrated farming systems is mainly due to interaction of multiple enterprises i.e., crops, livestock and poultry. Model M_7_ had obtained 116 and 133% higher gross and net returns, respectively compared to M_1_ model (Fig. 6). These results are supported by^22^ who found that gross and net income increased by 397 and 447%, respectively in integrated farming system compared to farmers practice. Higher B:C ratio was recorded in M_5_ followed by M_7_ and M_3_models because of profitable enterprises which results in higher returns. Although M_5_ has obtained higher B:C ratio, M_7_could be recommended to farmers because of higher net returns which is supported by^19,20^

### System economic efficiency

Models M_7_, M_5_ and M_3_have multiple profitable enterprises especially sheep and napier grass which has resulted in the higher system economic efficiency. These results are in agreement with^23^ who reported that crop + horticulture + diary + goat + poultry + vermicompost model has obtained highest system economic efficiency of ₹1257 day^−1^ followed by crop + horticulture + goat + poultry + vermicompost which has obtained ₹1118 day^−1^ because of integration of multiple profitable enterprises like goat and poultry.

Negi et al.^24^ reported that integrated farming systems obtains ₹353 ha^−1^ day^−1^ whereas conventional rice–wheat system obtains ₹132 ha^−1^ day^−1^ and integrated farming system obtains higher system economic efficiency because of integration of livestock. Models M_1_ and M_2_ have obtained low system economic efficiency as they have only cropping components. Existing cropping systems obtains low system economic efficiency and brings income once a year unlike farming systems which brings income round the year because of integration of multiple enterprises. These results are corroborated with^21^ who found that dependence on single component increases the risk to farmers especially with small holdings which necessitates the integration of cropping systems with livestock.

### Sustainable index

Identifying a suitable and viable IFS model for a region can be guided by a sustainability index, which indicates both economic viability and environmental friendliness. Higher sustainability index of models M_7_ and M_3_ is mainly because of integration of crop with livestock which provide continuous income. These results are in agreement with^23^ who found that livestock plays an important role in stabilizing the income sustainability if integrated with components like crop and horticulture. Babu et al. ^21^ reported that judicious integration and synergism among enterprises and efficient by-product utilization making the enterprises self-sustainable by generating wealth from waste which makes the system self sustainable. Swarnam et al.^12^ reported that cropping systems are vulnerable to abnormalities in this climate change era so animal components need to be integrated and diverse crops should be grown to achieve the sustainability to smallholder farms. Combining multiple productive and profitable components can improve the sustainability of a model. Therefore, promoting IFS can support marginal and small-scale farmers of Telangana^25^.

Negative sustainable index of models M_1_ and M_6_ was mainly due to a higher standard deviation of all the modules as compared to net returns or yield of that particular system. Ponnusamy et al.^26^ reported that adoption of only cropping components reduces the sustainable value index because of low net returns and diverse multiple components in a farming system enhance the sustainable yield as well as value index.

### Water footprints

Lowest water footprints were recorded in model M_7_ followed by M_3_ and M_5_ which might be because of havinglivestock components especially sheep which produce higher RGEY with less quantity of water compared to crops. M_1_ had obtained highest water footprints in both the years, respectively because of high water requirement of rice crop. Cereal crops are less water efficient but they are the staple food across the globe so they need to be integrated with other components to enhance overall water use efficiency of system. These results are in agreement with^15^ who reported that IFS unit has recorded the lowest water footprint of 149 lit per kg produce whereas conventional rice–wheat and maize–wheat systems have recorded 1277 and 1024 lit per kg produce, respectively which supports the notion that a suitable designed IFS module is water-use efficient under marginal land holding. Surve et al.^27^ reported that higher water productivity in the diversified systems was due to the multiple use of available water with the integration of different enterprises especillaycrop-livestock integration which is a regenerative agriculture practice which would benefit the farming community in the long term.

## Conclusions

Mono-cropping of cereals has been exploitative and not economical to the farmers. There is a need to design an agricultural production system that ensures food and nutritional security, provides social and economic stability, and builds and protects the ecosystem services. The IFS consists of different components viz., crops, perennials, fodder crops, livestock, poultry etc. that increases the productivity, profitability and employment which ultimately improves the standard of living of rural farmers. Farming systems reduces the dependence on external resources through efficient recycling of on-farm biomass and other resources. Conducting research on IFS helps to find out the contribution of each component and contribution to soil sustainability. Developing water efficient IFS model for small and marginal farmers is the need of the hour in this water scarce era. Model M_7_ has obtained higher system productivity and gross and net returns. It has recorded highest sustainable yield index and value index with lowest water footprints. Based on these results, it can be concluded that IFS model M_7_: Rice–Groundnut, Pigeonpea + Sweetcorn (1:3)—Bajra, Pigeonpea + Maize (1:3)—Sunhemp; Napier grass, Hedge lucerne, Poultry (100), Sheep (5 + 1) in 1-acre area is suitable for Irrigated Situation of Telangana and adjacent regions. Based on the results of this study, farmers and policy makers could understand the impact of integrated farming systems in terms of yields, profits and sustainability compared to traditional systems and work on increasing the adoption of this approach (Supplementary file).

### Supplementary Information

Supplementary Tables.

## Data Availability

The datasets used and/or analysed during the current study are available from the corresponding author on reasonable request.
