# Supplementary material for: Designing a productive, profitable integrated farming system model with low water footprints for small and marginal farmers of Telangana
Source: Sci Rep. 2024 Jul 24;14:17066. doi: 10.1038/s41598-024-66696-5 (PMC11269734; doi:10.1038/s41598-024-66696-5)
Supplement: Supplementary file 1 — Supplementary Tables. [file 41598_2024_66696_MOESM1_ESM.docx]

**Supplementary data – ANOVA Tables**

**Table 8a. ANOVA table of system productivity of all the IFS models in the year 2021-22**

| **Source of variation** | **Degrees of freedom** | **Sum of squares** | **Mean sum of squares** | **F cal** | **F prob/Table** |
| --- | --- | --- | --- | --- | --- |
| Replications | 2 | 229190 | 114595 | 0.338419 | 3.886 |
| Treatments | 6 | 60422906 | 10070484 | 29.73988 | 2.996 |
| Error | 12 | 4063426 | 338619 |  |  |
| Total | 20 |  |  |  |  |
| CD (p=0.05) | 1035 |  |  |  |  |
| CV | 9.35 |  |  |  |  |
| SEm(±) | 336 |  |  |  |  |

**Table 8b. ANOVA table of system productivity of all the IFS models in the year 2022-23**

| **Source of variation** | **Degrees of freedom** | **Sum of squares** | **Mean sum of squares** | **F cal** | **F prob/Table** |
| --- | --- | --- | --- | --- | --- |
| Replications | 2 | 316103 | 158052 | 0.419 | 3.885 |
| Treatments | 16 | 75655240 | 12609207 | 33.44 | 2.996 |
| Error | 12 | 4524223 | 377019 |  |  |
| Total | 20 |  |  |  |  |
| CD (p=0.05) | 1092 |  |  |  |  |
| CV | 9.08 |  |  |  |  |
| SEm(±) | 355 |  |  |  |  |

**Table 10a. ANOVA table of gross returns of all the IFS models in the year 2021-22**

| **Source of variation** | **Degrees of freedom** | **Sum of squares** | **Mean sum of squares** | **F cal** | **F prob/Table** |
| --- | --- | --- | --- | --- | --- |
| Replications | 2 | 31048689 | 15524344.5 | 0.161 | 3.886 |
| Treatments | 6 | 38480165134 | 6413360856 | **66.45** | 2.996 |
| Error | 12 | 1158127003 | 96510583.6 |  |  |
| Total | 20 |  |  |  |  |
| CD (p=0.05) | 17477 |  |  |  |  |
| CV | 7.53 |  |  |  |  |
| SEm(±) | 5672 |  |  |  |  |

**Table 10b. ANOVA table of net returns of all the IFS models in the year 2021-22**

| **Source of variation** | **Degrees of freedom** | **Sum of squares** | **Mean sum of squares** | **F cal** | **F prob/Table** |
| --- | --- | --- | --- | --- | --- |
| Replications | 2 | 15642915 | 7821458 | 0.324 | 3.886 |
| Treatments | 6 | 13092403584 | 2182067264 | **90.60** | 2.996 |
| Error | 12 | 289024744 | 24085395 |  |  |
| Total | 20 |  |  |  |  |
| CD (p=0.05) | 8731 |  |  |  |  |
| CV | 6.95 |  |  |  |  |
| SEm(±) | 2833 |  |  |  |  |

**Table 10c. ANOVA table of B:C ratio of all the IFS models in the year 2021-22**

| **Source of variation** | **Degrees of freedom** | **Sum of squares** | **Mean sum of squares** | **F cal** | **F prob/Table** |
| --- | --- | --- | --- | --- | --- |
| Replications | 2 | 0.0082 | 0.0041 | 0.201 | 3.886 |
| Treatments | 6 | 0.189 | 0.0315 | **1.543** | 2.996 |
| Error | 12 | 0.244976 | 0.020415 |  |  |
| Total | 20 |  |  |  |  |
| CD (p=0.05) | 0.254 |  |  |  |  |
| CV | 6.614 |  |  |  |  |
| SEm(±) | 0.082 |  |  |  |  |

**Table 10d. ANOVA table of gross returns of all the IFS models in the year 2022-23**

| **Source of variation** | **Degrees of freedom** | **Sum of squares** | **Mean sum of squares** | **F cal** | **F prob/Table** |
| --- | --- | --- | --- | --- | --- |
| Replications | 2 | 3969569 | 1984785 | 0.0182 | 3.886 |
| Treatments | 6 | 31486043079 | 5247673846 | **48.107** | 2.996 |
| Error | 12 | 1308976728 | 109081394 |  |  |
| Total | 20 |  |  |  |  |
| CD (p=0.05) | 18580 |  |  |  |  |
| CV | 7.57 |  |  |  |  |
| SEm(±) | 6030 |  |  |  |  |

**Table 10e. ANOVA table of net returns of all the IFS models in the year 2022-23**

| **Source of variation** | **Degrees of freedom** | **Sum of squares** | **Mean sum of squares** | **F cal** | **F prob/Table** |
| --- | --- | --- | --- | --- | --- |
| Replications | 2 | 19504339 | 9752169 | 0.273 | 3.886 |
| Treatments | 6 | 16426309587 | 2737718264 | **76.73** | 2.996 |
| Error | 12 | 428141895 | 35678491 |  |  |
| Total | 20 |  |  |  |  |
| CD (p=0.05) | 10626 |  |  |  |  |
| CV | 7.169 |  |  |  |  |
| SEm(±) | 3449 |  |  |  |  |

**Table 10f. ANOVA table of B:C ratio of all the IFS models in the year 2022-23**

| **Source of variation** | **Degrees of freedom** | **Sum of squares** | **Mean sum of squares** | **F cal** | **F prob/Table** |
| --- | --- | --- | --- | --- | --- |
| Replications | 2 | 0.0122 | 0.0061 | 0.193 | 3.885 |
| Treatments | 6 | 1.687 | 0.2812 | **8.909** | 2.996 |
| Error | 12 | 0.379 | 0.032 |  |  |
| Total | 20 |  |  |  |  |
| CD (p=0.05) | 0.316 |  |  |  |  |
| CV | 7.106 |  |  |  |  |
| SEm(±) | 0.1026 |  |  |  |  |

**Table 11a. ANOVA table of water footprints of all the IFS models in the year 2021-22**

| **Source of variation** | **Degrees of freedom** | **Sum of squares** | **Mean sum of squares** | **F cal** | **F prob/Table** |
| --- | --- | --- | --- | --- | --- |
| Replications | 2 | 2278.31 | 1139.16 | 0.753 | 3.885 |
| Treatments | 6 | 521989.78 | 86998.30 | **57.51** | 2.996 |
| Error | 12 | 18152.815 | 1512.73 |  |  |
| Total | 20 |  |  |  |  |
| CD (p=0.05) | 69.19 |  |  |  |  |
| CV | 7.464 |  |  |  |  |
| SEm(±) | 22.455 |  |  |  |  |

**Table 11b. ANOVA table of water footprints of all the IFS models in the year 2022-23**

| **Source of variation** | **Degrees of freedom** | **Sum of squares** | **Mean sum of squares** | **F cal** | **F prob/Table** |
| --- | --- | --- | --- | --- | --- |
| Replications | 2 | 2399.66 | 1199.83 | 1.180 | 3.885 |
| Treatments | 6 | 476180.21 | 79363.36 | **78.09** | 2.996 |
| Error | 12 | 12195.09 | 1016.25777 |  |  |
| Total | 20 |  |  |  |  |
| CD (p=0.05) | 56.71 |  |  |  |  |
| CV | 7.348 |  |  |  |  |
| SEm(±) | 18.405 |  |  |  |  |
